# No Relationship between Lean Mass and Functional Asymmetry in High-Level Female Tennis Players

**DOI:** 10.3390/ijerph182211928

**Published:** 2021-11-13

**Authors:** Laurent Chapelle, Chris Bishop, Peter Clarys, Eva D’Hondt

**Affiliations:** 1 Department of Movement and Sport Sciences, Faculty of Physical Education and Physiotherapy, Vrije Universiteit Brussel, 1050 Brussels, Belgium; peter.clarys@vub.be (P.C.); eva.dhondt@vub.be (E.D.); 2 London Sport Institute, Middlesex University, London NW4 4BT, UK; c.bishop@mdx.ac.uk; 3 Department of Movement and Sports Sciences, Faculty of Medicine and Health Sciences, Ghent University, 9000 Ghent, Belgium

**Keywords:** women, performance, unilateral, racket sport

## Abstract

The relationship between lean mass and functional asymmetry in terms of their magnitude and direction was examined in 22 high-level female tennis players (20.9 ± 3.6 years). Lean mass of both upper and lower extremities was examined using Dual X-ray Absorptiometry. Functional asymmetry was assessed using a battery of field tests (handgrip strength, seated shot-put throw, plate tapping, single leg countermovement jump, single leg forward hop test, 6 m single leg hop test, and 505 change of direction (time and deficit)). Paired sample *t*-tests compared the dominant (overall highest/best (performance) value) against the non-dominant value (highest/best (performance) value of the opposing extremity). Linear regressions were used to explore the relationship between lean mass and functional asymmetry magnitudes. Kappa coefficients were used to examine the consistency in direction between the extremity displaying the highest lean mass value and the extremity performing dominantly across tests. Significant asymmetry magnitudes (*p* < 0.05) were found for all upper and lower extremity lean mass and functional values. No relationship was apparent between lean mass and functional asymmetry magnitudes (*p*-value range = 0.131–0.889). Despite finding perfect consistency in asymmetry direction (k-value = 1.00) for the upper extremity, poor to fair consistency (k-value range = −0.00–0.21) was found for the lower extremity. In conclusion, lean mass and functional asymmetries should be examined independently.

## 1. Introduction

As one of the most popular sports globally, tennis is characterised by short high-intensity efforts which are alternated by bouts of recovery [1,2]. During these high-intensity efforts, tennis strokes are performed during which the preferred upper extremity of the player (i.e., the upper extremity holding the racket) is exposed to greater mechanical loading compared to the opposing upper extremity (i.e., the non-preferred upper extremity) [3]. Consequently, this predominantly unilateral sport is ideally suited to examine the occurrence of lean mass asymmetries (i.e., side-to-side differences in lean mass, expressed as a percentage) [4,5]. For instance, using Dual X-ray Absorptiometry (DXA), significant asymmetries between the preferred and non-preferred upper extremity in terms of lean mass (i.e., which includes muscle mass and body water) have previously been reported in both male (i.e., 9.7%) and female (i.e., 6.8%) tennis players [6,7].

In addition to the upper limbs, the lower extremities of tennis players are also subjected to asymmetrical loading due to their specific role in the kinetic chain when performing the various tennis strokes [8,9]. Several previous studies have examined lower extremity lean mass asymmetries by means of DXA in male youth [10], professional male adult [11] and high-level female adult tennis players [6], but reported varying results. For instance, the two beforementioned studies examining (youth) male tennis players indicated no significant lower extremity lean mass asymmetries (i.e., 0.6–0.8%), whilst the study examining female tennis players demonstrated significant lower extremity lean mass asymmetries (i.e., 4.8%). An important consideration, however, is that the latter study did not relate these significant side-to-side differences in lower extremity lean mass to players’ tennis-specific physical performance (which may increase our knowledge regarding the impact of lean mass asymmetries). Hence, and in addition to the reported contradictory results, more research into (lower extremity) lean mass asymmetries in female tennis players is warranted.

Along with the occurrence of lean mass asymmetry, the presence of functional asymmetry (i.e., side-to-side differences in physical performance (e.g., strength or power), again expressed as a percentage) has also been established. Consequently, significant magnitudes of upper (i.e., 8.9–15.2%) and lower extremity (i.e., 1.8–9.4%) functional asymmetries have previously been reported in high-level female tennis players [12]. When examining functional asymmetries, it is essential to use a composite test battery (as opposed to isolated testing) given the direction specificity of asymmetries (i.e., which extremity displays higher values and/or is dominant in performance) between different sporting tasks [13]. For instance, the beforementioned study in high-level female tennis players reported that the preferred upper extremity consistently demonstrates superior performances compared to the opposing upper extremity. In contrast, the lower extremity was found to display poor levels of agreement as to which leg performed better across tests (i.e., the kappa coefficients ranged from −0.07 to 0.17), illustrating the direction specificity of lower extremity functional asymmetries [12].

It is important to note that both lean mass and functional asymmetries have, albeit separately, been associated with a decreased sport-specific performance, in addition to an increased injury risk [13,14,15]. However, no study has simultaneously examined both types of asymmetry. As a result, research regarding the relationship between lean mass asymmetry and functional asymmetry, both at the upper and lower extremity level, is currently lacking. More specifically, it is unknown whether a high(er) magnitude of lean mass asymmetry implies a high(er) magnitude of functional asymmetry (i.e., which could be the case since muscle mass (which entails lean mass) is reported to be a key determinant of functional strength and power) [16]. Similarly, regarding the agreement in direction between lean mass and functional asymmetry, it is unknown whether the extremity that displays the highest lean mass value also displays the best performance across body sides. As a result, the mutual relationship and the agreement in direction between both lean mass asymmetry and functional asymmetry remains to be investigated. Due to the lack of previous research in this respect (especially in female tennis players), this study aimed to examine the relationship between lean mass and functional asymmetry in terms of their magnitude and direction in high-level female tennis players.

## 2. Materials and Methods

### 2.1. Participants

Twenty-two high-level Belgian female tennis players aged between 17 and 27 years participated in this observational cross-sectional study. To be eligible for participation, these female tennis players had to be injury-free at the time of measurement and either have an international tennis ranking (i.e., Women’s Tennis Association or International Tennis Federation) or a high national tennis ranking (i.e., being in the top 200 of the Belgian circuit ranking). Our study protocol was approved by the local university’s medical ethics committee prior to data collection (B.U.N. 143201836107). The female tennis players, together with their legal guardians if they were still minor, were informed about the purposes of this study and signed an informed consent upon participation.

### 2.2. Procedures and Experimental Design

Data collection took place in the local university’s biometry and biomechanics laboratory facilities between March 2019 and September 2020. Firstly, the female tennis players were asked to fill in a questionnaire to provide basic demographic and sport-specific information (i.e., date of birth, dominant upper extremity, starting age of tennis play and average weekly training volume over the last year). Next, after voiding their bladder and whilst being barefoot in light sports clothing, participants’ body height and weight were measured to the nearest 0.1 cm and 0.002 kg using a stadiometer (SECA 217, Hamburg, Germany) and precision scale (RADWAG WLT 60/120/X/L3, All scales Europe, Veen, The Netherlands), respectively. Table 1 presents the demographic, sport-specific and anthropometric information of the 22 female tennis players included in our study sample.

### 2.3. Lean Mass

DXA research scans (Norland Elite, Swissray, Fort Atkinson, WI, USA) of both the preferred and non-preferred upper extremity as well as the right and left lower extremity were conducted by the same researcher, who was intensively trained by the DXA scan manufacturer upon data collection, in order to determine participants’ regional lean mass to the nearest 0.1 g. The DXA scanner was calibrated in accordance with the manufacturer’s guidelines before each test session. Participants were instructed to lie as straight and still as possible in a supine position on the DXA scan table after the removal of all metal objects (e.g., earrings). The scan width was set to 6 × 6 mm, whilst a scan speed of 130 mm/s was applied. The upper extremity region included the upper arm, lower arm and hand, and was separated from the trunk by an inclined line passing through the scapula-humeral joint. The lower extremity region included the upper leg, lower leg and foot, and was separated from the trunk by an inclined line passing just below the pelvis [11]. The DXA research scans were analysed with the Norland Illuminatus software (Swissray, Fort Atkinson, WI, USA).

### 2.4. Functional Test Battery

A physical performance field-based test battery was used to examine the magnitude of functional asymmetry. Participants were instructed to wear their normal tennis outfit and sports shoes whilst performing the test battery, consisting of 8 different unilateral tests. A standardised 10-min warm-up, involving light running exercises and dynamic stretches, was implemented before completing the test battery. The different tests were always completed in the same order, ensuring alternation in testing the upper and lower extremities. The participants were guided through the test battery by the same well-trained researcher. Each participant was given three attempts per body side for every test. The first attempt of a test was always performed with the right body side, whereas the second attempt was always performed with the left body side, ensuring alteration between both sides of the body during testing. Participants were given 60 s of rest between attempts and 3 min of rest between tests to ensure adequate recovery.

Handgrip strength: Participants were instructed to squeeze as hard as possible (for three seconds) in a digital handheld dynamometer with an accuracy of 0.1 kg (Jamar Plus, Patterson Medical, Nottinghamshire, UK), while being seated in a chair without armrests. The elbow of the participants had to remain 90 degrees flexed throughout every attempt [17].

Seated shot-put throw: Participants were seated on the ground with their back against a wall and their hips, knees and ankles parallel to the ground. The non-throwing arm was placed on the opposite (i.e., throwing) shoulder. From this position, participants had to throw a 3-kg medicine ball as far as possible in a forward direction. The distance where the medicine ball landed on to the ground was measured to the nearest 1 cm using a tape measure [18].

Plate tapping: Two discs (with a diameter of 20 cm) were placed with their centres 60 cm apart on a table together with a 10 × 20 cm rectangle (which was placed in between the two discs). Participants started the test with one hand placed on one of the two discs, whilst the other hand was placed on the rectangle in the middle. The aim of the plate tapping test was to move one hand back and forth between both discs over the other hand (which was on the rectangle) as fast as possible. This action was repeated for 25 full cycles (i.e., 50 taps on the discs) and the time needed to complete this test was recorded to the nearest 0.01 s using a hand-held stopwatch [19].

Single leg countermovement jump: Participants were instructed to jump up as high as possible on one leg. Throughout the jump, they were instructed to hold their hands on their hips. Swinging of the non-jumping leg was not allowed and the jumping leg had to remain completely extended throughout the flight phase. Participants needed to keep their balance on one leg after landing, otherwise an extra attempt was provided. Jumping height was determined to the nearest 0.1 cm using the Optojump Next system (Microgate Bolzano, Italy) [20].

Single leg forward hop test: Participants stood on one leg behind a tape line whilst holding their hands on the hips. They had to jump as far as possible in a forward direction landing on the same foot without losing their balance (e.g., moving their foot on which they land or planting the other foot on to the ground). If participants were not able to maintain their balance on one leg after landing, an extra attempt was provided. The covered distance from the starting line to the heel of the participants’ landing foot was measured to the nearest 1 cm using a tape measure [21].

6 m single leg hop test: Participants were instructed to cover 6 m as fast as possible whilst hopping on one leg. The time needed to cover these 6 m was measured to the nearest 0.001 s using electronic timing gates (Witty Wireless Training Timer, Microgate, Bolzano, Italy). These timing gates were placed at hip height and participants had to start behind a tape line which was located 30 cm from the first timing gate.

505 change of direction time (505 COD time) and deficit (505 COD deficit): First, participants’ 10 m sprint time was measured to the nearest 0.001 s using electronic timing gates (Witty Wireless Training Timer, Microgate, Bolzano, Italy). Next, their 505 COD time was determined to the nearest 0.001 s based on performing the 505 COD test, which consisted of a 5 m sprint, followed by a 180° turn to either the left or the right side, and a 5 m sprint back to the starting line. Participants’ 505 COD deficit was then calculated by deducting their 10 m sprint time from their 505 COD time [22].

### 2.5. Asymmetry Calculations

The dominant value was defined as the highest lean mass value or the best (i.e., highest or fastest) value for a test of the functional test battery. The non-dominant value was defined as the highest or best result of the same outcome measure for the opposing upper or lower extremity [23]. The magnitude of lean mass and functional asymmetry was calculated for every outcome measure and expressed as a percentage by using the percentage difference method (PDM): (dominant value − non-dominant value)/dominant value) × 100 [24].

### 2.6. Statistical Analyses

Data analysis was conducted using SPSS version 27.0 (IBM, Chicago, IL, USA). Normality of distribution was examined for every outcome measure using the Shapiro–Wilk test. Variability and reliability of every outcome measure was verified by calculating the coefficient of variation (CV) and a two-way random intraclass correlation coefficient (ICC) with 95% confidence intervals. CV values of less than 10% were considered acceptable and ICC values were classified as poor (<0.50), moderate (0.50–0.74), good (0.75–0.89) and excellent (>0.90) [25,26]. Paired sample *t*-tests were used for within-subject comparisons of the dominant against the non-dominant values for every outcome measure. Effect size analyses using Hedges’ *g* were conducted of the side-to-side difference between the dominant and non-dominant values and classified as trivial (<0.20), small (0.20–0.49), medium (0.50–0.79) or large (>0.80) [27]. A linear regression analysis, adjusting for the participants’ age, was used to examine the relationship between the magnitude of lean mass asymmetry and the magnitude of functional asymmetry [28]. Lastly, the consistency in direction as to which extremity displayed the dominant lean mass value and which extremity performed dominantly across the different field tests of the functional test battery was examined using Kappa coefficients. These Kappa coefficients were classified as poor (≤0), slight (0.01–0.20), fair (0.21–0.40), moderate (0.41–0.60), substantial (0.61–0.80), almost perfect (0.81–0.99) and perfect (1.00) [29]. All data are presented as means ± standard deviations and *p*-values <0.05 were considered statistically significant.

## 3. Results

Every outcome measure showed acceptable reliability (i.e., all CVs were below 10%) and excellent reliability (i.e., all ICCs were above 0.90) as presented in Table 2. The lean mass and functional asymmetry values for our study sample of high-level female tennis players are displayed in Table 3. Significant magnitudes of lean mass and functional asymmetry for all outcome measures were found (t-value range = 4.027–8.638; *p* < 0.001). Effect sizes between the side-to-side differences of the dominant and non-dominant values ranged from small to large.

**Figure 1:**
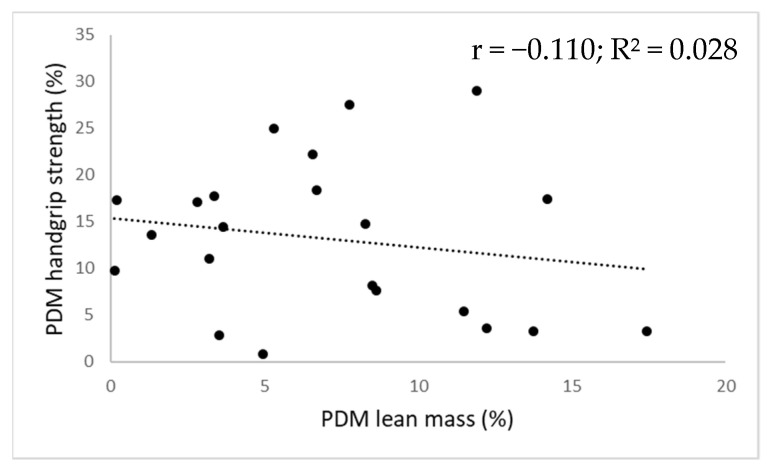

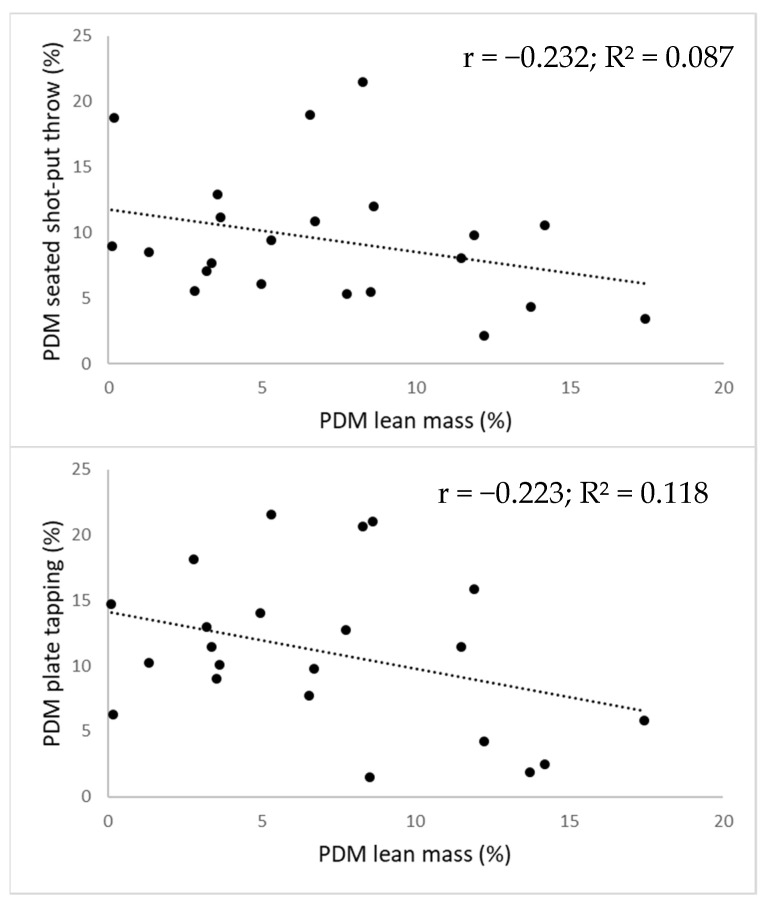
Scatter plot illustrating the relationship between the magnitude of upper extremity lean mass asymmetry (x-axis) and the magnitude of upper extremity functional asymmetry (y-axis) for the high-level female tennis players (N = 22). Note: The dotted line represents the linear trend line; PDM = percentage difference method; r = correlation coefficient; R² = R squared value.

**Figure 2:**
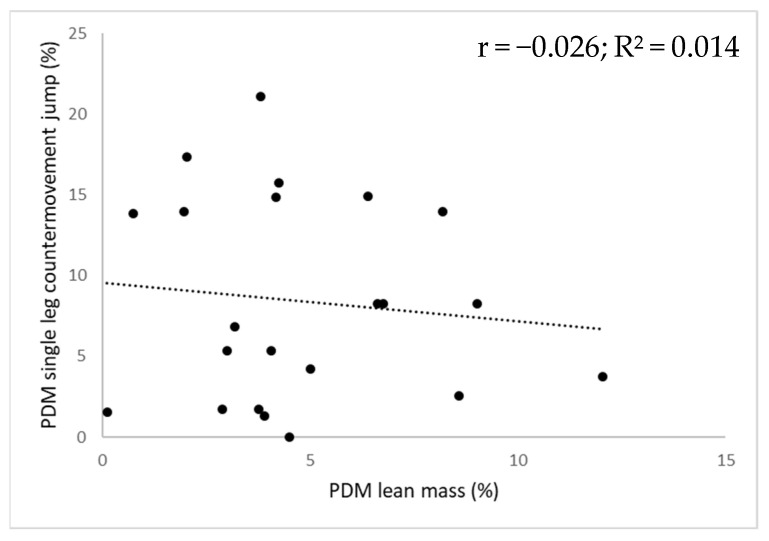

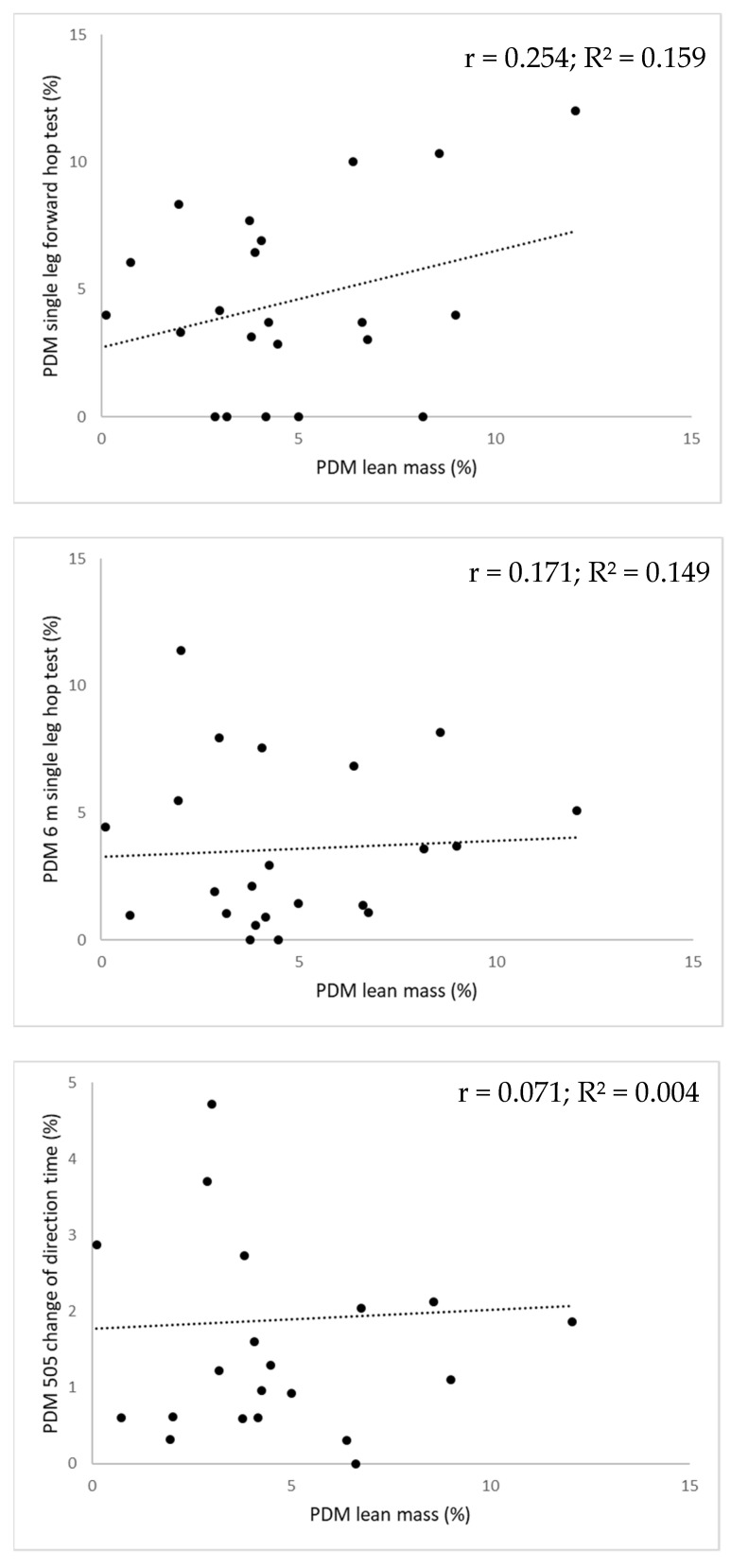

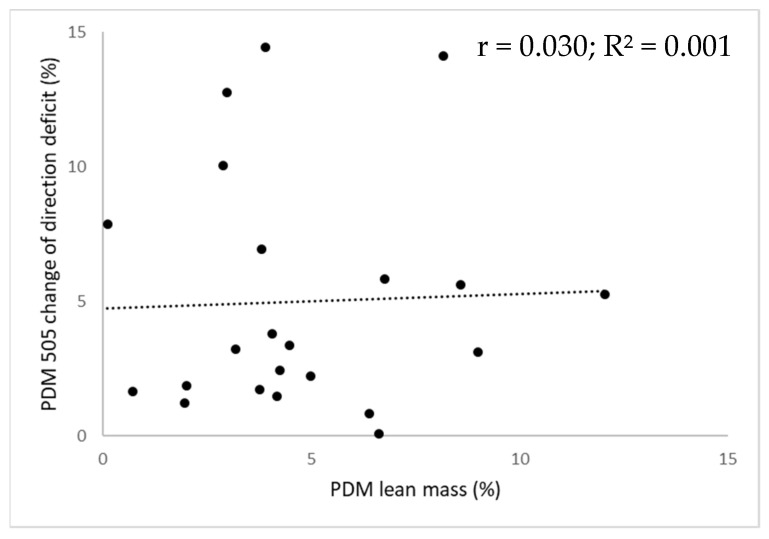
Scatter plot illustrating the relationship between the magnitude of lower extremity lean mass asymmetry (x-axis) and the magnitude of lower extremity functional asymmetry (y-axis) for the high-level female tennis players (N = 22). Note: The dotted line represents the linear trend line; PDM = percentage difference method; r = correlation coefficient; R² = R squared value.

For every field-based test, the corresponding individual lean mass asymmetry magnitudes alongside functional asymmetry magnitudes are displayed in Figure 1 for the upper extremity and in Figure 2 for the lower extremity. No significant relationship between the magnitude of lean mass asymmetry and the magnitude of functional asymmetry (F-value range = 0.021–3.461; r-value range = −0.232–0.254; *p*-value range = 0.131–0.889) was found as lean mass asymmetry magnitude could only explain 0.1 to 15.9% of the functional asymmetry magnitude.

The consistency in direction between the upper extremity displaying the dominant lean mass value and the upper extremity performing dominantly on the tests of the functional test battery was classified as perfect. For the lower extremity, the consistency between the lower extremity displaying the dominant lean mass value and the lower extremity performing dominantly across tests were classified from poor to fair (Table 4).

The consistency in direction between the upper extremity displaying the dominant lean mass value and the upper extremity performing dominantly on the tests of the functional test battery was classified as perfect. For the lower extremity, the consistency between the lower extremity displaying the dominant lean mass value and the lower extremity performing dominantly across tests was classified from poor to fair, depending on the field test at hand (Table 4).

## 4. Discussion

This observational cross-sectional study aimed to examine the relationship between lean mass and functional asymmetry in terms of their magnitude and direction in high-level female tennis players. The results of our study indicated no meaningful relationships between the magnitude of lean mass asymmetry and functional asymmetry in either the upper or the lower extremities. Additionally, consistency in the direction of asymmetry between the extremity displaying the highest lean mass value and the extremity displaying the dominant performance value for the functional tests across body sides was perfect for the upper extremity, whereas this consistency in dominance for both types of asymmetry ranged from poor to fair as regards to the lower extremity.

The significant magnitude of upper extremity lean mass asymmetry found in this study (i.e., 7.1%) can be largely attributed to the mechanical loading imposed to the preferred upper extremity associated with the repetitive performance of tennis strokes [4]. Interestingly, the preferred upper extremity of all high-level female tennis players included in the present always displayed the highest lean mass value. In agreement with the results of the upper extremity, significant lower extremity lean mass asymmetries (i.e., 4.8%) were found in our sample of Belgian high-level female tennis players. Even though most of them were right-handed (i.e., 21 out of 22 players), the majority displayed a higher lean mass of the left leg compared to the right leg (i.e., 18 out of 22 players). This could be explained by the previously reported occurrence of cross-asymmetry where the contralateral leg (i.e., the leg opposed to the preferred upper extremity) plays an important role in counterbalancing the torques of the upper extremity performing the various tennis strokes [6,8,9]. It is important to consider that the present study compared the dominant versus the non-dominant value to examine and report lower extremity lean mass asymmetries as opposed to using the values of the self-reported preferred lower extremity by asking, for example, on which leg participants prefer to perform a single leg hop [24]. The latter could lead to an incorrect calculation of the asymmetry magnitude as a percentage should be calculated with respect to the highest value [24,30].

The magnitude of upper extremity functional asymmetry ranged from 9.5 to 13.2% in our study, which is indicative of significant inter-limb asymmetries. Again, these significant inter-limb asymmetries can be principally attributed to the predominantly unilateral nature of tennis [3]. It is important to note that the preferred upper extremity of the included high-level female tennis players always performed dominantly across all upper extremity tests. Although lower than the magnitude of upper extremity functional asymmetry, the overall magnitude of functional asymmetry at the lower extremity level ranged from 1.9 to 8.4%, indicating significant functional asymmetries for all lower extremity performance tests. However, due to the task specificity of lower extremity functional asymmetries, there was no occurrence of cross-asymmetry across the functional tests for the lower extremity, as also mentioned in earlier research [12]. The highest asymmetry magnitude was found for the single leg countermovement jump (i.e., 8.4%). This result is in agreement with previous studies that have reported jump height from the single leg countermovement jump as being a sensitive physical performance test to examine functional asymmetries, especially when compared to jumping in a forward direction [31,32]. Nevertheless, it can be argued that it is surprising to find significant lower extremity functional asymmetries in a study sample of high-level female tennis players because being equally physically skilled on both lower extremities could be advantageous from a performance perspective [13].

As indicated by the results of this study, lean mass asymmetry and functional asymmetry do not seem to be related in terms of their magnitude given that lean mass asymmetry magnitude could only explain between 0.1 and 15.9% of the functional asymmetry magnitude. This is surprising because lean mass (which also encompasses muscle mass) has been reported to be a key determinant of functional strength and power [16], although it has been reported that other factors such as neuromuscular control and joint coordination also contribute to strength and power development [15,33]. Therefore, it is recommended that practitioners examine lean mass and functional asymmetries independently from one another. Additionally, the non-existent relationship between lean mass and functional asymmetry may have implications when designing targeted training programmes to counteract the reported negative influences of asymmetry (as it is unclear whether practitioners should focus on lean mass and/or functional parameters) [13,14,15]. Regarding the direction of asymmetry, there was a poor to slight consistency between the lower extremity displaying the dominant lean mass value and the lower extremity performing dominantly across the functional tests. This result was in contrast to the upper extremity, which displayed perfect levels of agreement. Consequently, the reported lower extremity results in this respect highlight the task and direction specific nature of asymmetry during the execution of different tasks, with Kappa values of the present study being comparable to those in previous research [20,32]. Because the extremity displaying the highest lean mass value does not consistently perform dominantly at lower limb level, it is recommended that practitioners examine and interpret both lean mass and functional asymmetry in an independent manner. Additionally, the assessment of asymmetries should be performed regularly and on an individual player basis, so that an asymmetry profile can be made to closely monitor each tennis player [20,34].

This is the first study to examine and report both lean and functional asymmetry of the upper and the lower extremity in high-level female tennis players using individual data. It can be argued that high-level tennis players are well suited to examine asymmetries because reaching such a level requires a high training volume and given the reported association between a high training volume and the occurrence of asymmetry [10]. Additionally, all players included in our study sample started to play tennis before the onset of puberty, which has been reported to result in greater asymmetry magnitudes [4]. Furthermore, functional asymmetry was examined using a valid, reliable and elaborated field-based test battery, as opposed to isolated testing, which is important given that asymmetries are reported to be movement or task-specific [34], as clearly demonstrated by our findings at the level of the lower limb. However, some limitations to our research are apparent. The present study implemented a cross-sectional design, which included a small sample size (although a post hoc power analysis revealed that the statistical power of this study was 91%). However, a control group was not included and the association between lean mass and functional asymmetry with decreased sport-specific performance, and injury incidence, was not examined. Therefore, future research is needed to examine the influence of lean mass and functional asymmetry on sports-specific performance and injury incidence using a longitudinal design. Additionally, more precise tools (e.g., force plates or isokinetic dynamometry) and outcome measures (e.g., leg stiffness, ground contact time or force) could be used when examining functional asymmetries [35].

## 5. Conclusions

To conclude, the significant lean mass and functional asymmetries of both the upper and lower extremity were not related in terms of their magnitude among high-level female tennis players. Additionally, the consistency between the extremity displaying the dominant lean mass value and the extremity displaying the dominant performance value across the functional tests was perfect for the upper extremity, whereas this consistency ranged from poor to fair for the lower extremity. When examining asymmetries in tennis players, it is recommended that both the magnitude and direction thereof should be considered and interpreted independently of one another in view of asymmetry profiling because no mutual relationship between both constructs could be demonstrated. It is also essential to examine and monitor both upper and lower extremity asymmetries on an individual player basis and to examine functional asymmetries using an elaborated field-based test battery. Future more in-depth research is also needed to investigate the impact of lean mass and functional asymmetries on female players’ sports-specific performance and injury incidence using longitudinal (and/or experimental) study designs.

## Figures and Tables

**Table 1:** Demographic, sport-specific and anthropometric information of the high-level female tennis players (N = 22).

	High-Level Female Tennis Players
Age (years)	20.9 ± 3.6
Height (cm)	169.5 ± 4.8
Weight (kg)	62.5 ± 8.3
Starting age of tennis play (years)	6.1 ± 1.4
Training volume (hours/week)	10.2 ± 6.2
Handedness (n, right/left)	21/1

Note: Data are presented as n or mean ± standard deviation.

**Table 2:** Variability and reliability of the DXA research scans and the unilateral tests of the functional test battery.

	CV	ICC (95% CI)
DXA research scan		
Upper extremity lean mass	2.3	0.99 (0.99, 1.00)
Lower extremity lean mass	2.3	0.98 (0.98, 1.00)
Functional test battery		
Upper extremity field tests		
Handgrip strength	2.9	0.96 (0.95, 0.98)
Seated shot-put throw	4.8	0.95 (0.91, 0.98)
Plate tapping	3.8	0.94 (0.89, 0.97)
Lower extremity field tests		
Single leg countermovement jump	5.9	0.97 (0.95, 1.00)
Single leg forward hop test	3.5	0.98 (0.96, 1.00)
6 m single leg hop test	2.4	0.98 (0.95, 1.00)
505 changes of direction		
Time	1.5	0.98 (0.96, 1.00)
Direction	3.9	0.97 (0.92, 1.00)

Note: DXA = Dual X-ray Absorptiometry; CV = coefficient of variation; ICC = intraclass correlation coefficient; 95% CI = 95% confidence interval.

**Table 3:** Upper and lower extremity lean mass and functional asymmetry values of the high-level female tennis players (N = 22).

	Dominant Value	Non-Dominant Value	ES (95% CI)	PDM (%)
Lean mass				
Upper extremity (g)	2069.9 ± 356.8	1935.1 ± 299.1	0.41 (−0.10, 0.90)	7.1 ± 4.8 *
Lower extremity (g)	8453.8 ± 1226.0	8060.7 ± 1225.8	0.31 (−0.18, 0.81)	4.8 ± 2.9 *
Functional test battery				
Upper extremity				
Handgrip strength (kg)	38.9 ± 6.7	33.8 ± 5.8	0.80 (0.28, 1.31)	13.2 ± 8.3 *
Seated shot-put throw (cm)	328.2 ± 45.9	296.8 ± 44.0	0.70 (0.18, 1.20)	9.5 ± 5.0 *
Plate tapping (sec)	10.24 ± 1.50	11.47 ± 1.75	0.74 (0.23, 1.25)	11.1 ± 6.0 *
Lower extremity				
Single leg countermovement jump (cm)	15.0 ± 3.5	13.7 ± 3.0	0.40 (−0.11, 0.89)	8.4 ± 6.3 *
Single leg forward hop test (cm)	142.7 ± 16.7	136.1 ± 18.5	0.37 (−0.13, 0.87)	4.8 ± 4.2 *
6 m single leg hop test (sec)	1.938 ± 0.168	2.010 ± 0.172	0.42 (−0.09, 0.84)	3.6 ± 3.2 *
505 change of direction				
Time (sec)	3.249 ± 0.174	3.311 ± 0.181	0.34 (−0.16, 0.84)	1.9 ± 1.7 *
Deficit (sec)	1.144 ± 0.109	1.207 ± 0.120	0.54 (−0.03, 1.04)	5.0 ± 4.3 *

Note: Data are presented as mean ± standard deviation; ES = effect size; 95% CI = confidence interval; PDM = percentage difference method; * Significant (*p* < 0.05) magnitude of asymmetry between body sides.

**Table 4:** Kappa coefficients indicating the consistency in direction between the dominant lean mass value and the dominant performance value across unilateral tests for the high-level female tennis players (N = 22).

	Kappa	Description
Upper extremity lean mass		
Handgrip strength	1.00	Perfect
Seated shot-put throw	1.00	Perfect
Plate tapping	1.00	Perfect
Lower extremity lean mass		
Single leg countermovement jump	0.18	Slight
Single leg forward hop test	0.00	Poor
6 m Single leg hop test	0.18	Slight
505 Change of direction time/deficit	0.21	Fair

Note: Kappa coefficients are classified as poor (≤0), slight (0.01–0.20), fair (0.21–0.40), moderate (0.41–0.60), substantial (0.61–0.80), almost perfect (0.81–0.99) and perfect (1.00).

## Data Availability

The data that support the findings of this study are available from the corresponding author upon request.
